# Effect of cannabis smoking on lung function and respiratory symptoms: a structured literature review

**DOI:** 10.1038/npjpcrm.2016.71

**Published:** 2016-10-20

**Authors:** Luis IG Ribeiro, Philip W Ind

**Affiliations:** 1 Department of Medicine, Imperial College London, London, UK; 2 Respiratory Medicine, National Heart and Lung Institute, Imperial College London, Hammersmith Hospital, Hammersmith Campus, London, UK

## Abstract

As cannabis use increases, physicians need to be familiar with the effects of both cannabis and tobacco on the lungs. However, there have been very few long-term studies of cannabis smoking, mostly due to legality issues and the confounding effects of tobacco. It was previously thought that cannabis and tobacco had similar long-term effects as both cause chronic bronchitis. However, recent large studies have shown that, instead of reducing forced expiratory volume in 1 s and forced vital capacity (FVC), marijuana smoking is associated with increased FVC. The cause of this is unclear, but acute bronchodilator and anti-inflammatory effects of cannabis may be relevant. Bullous lung disease, barotrauma and cannabis smoking have been recognised in case reports and small series. More work is needed to address the effects of cannabis on lung function, imaging and histological changes.

## Introduction

As most people are aware, marijuana is the most widely used illicit drug in the world.^1^ Cannabis is the second most smoked substance, after tobacco. In the past few years, recreational use of cannabis (‘weed’, ‘dope’, ‘grass’, ‘spliff’, ‘toke’, ‘hash’, ‘hemp’, ‘bud’, ‘ganja’ and many others) has had increasing media attention and, with legislation in various countries being relaxed, it appears likely that there will be an increase in exposure generally. However, we still know very little about the long-term effects of smoking cannabis on the respiratory system and on health in general.

Cannabis comes from a flowering plant, native to central Asia and the Indian sub-continent. The genus includes three different species—*Cannabis sativa*, *Cannabis indica* and *Cannabis ruderalis*. They produce two major active compounds, delta-9-tetrahydrocannabinol (d-9-THC) and cannabidiol (CBD); however, they contain 60 cannabinoids and over 400 compounds in total.^2^ THC is the psychoactive compound, but it is modulated by CBD. The *C. sativa*-dominant strains have higher THC content, whereas the *C. indica*-dominant strains have higher CBD content.^2^ The *C. ruderalis*-dominant strains contain even lower THC concentrations than the *C. indica-*dominant strains.^3^ There is also ‘skunk-like’ cannabis, named for its smell, containing very high THC concentrations.^4^

Experimentally, the acute bronchodilator effect^5,6^ and analgesic properties^7–9^ of inhaled cannabis are well described. Acute bronchodilatation is due to THC.^4^ However, as cannabinoids can have partial agonist, or even antagonist, effects, little is known about differences in airway effects from different strains of cannabis containing varying concentrations of cannabinols.

Evidence that chronic cannabis users have an increased incidence of respiratory symptoms such as chronic cough, sputum production, dyspnoea, hoarse voice and chest tightness has been reviewed recently.^6,10^ As marijuana smoke contains many of the same compounds as, and shares similar properties with, cigarette smoke, respiratory symptoms would be expected.^11,12^

### Methodological difficulties

There are obvious inherent difficulties in studying the long-term use of illicit substances. Examining dose–response is confounded by the problem of quantifying cannabis use—the measurement of ‘joint-years’ has inherent difficulties. One joint-year is defined as 365 joints smoked. However, because of the wide variety and strengths of marijuana in a joint,^13,14^ as well as the varying amounts that individuals put into a joint, this measure differs markedly between individuals. Reproducibility of joints in the same individual is not well defined. Furthermore, most studies use self-reported joint-years to quantify use; this may be inaccurate owing to the legality of cannabis use, recall bias and alteration of behaviour by its observation.

Another major consideration is the variety of ways that cannabis can be smoked, which alter the characteristics of the smoke inhaled. Using a water bong, e.g., decreased the concentration of inhaled carcinogenic compounds.^11,15^ Recent evidence has suggested that using a vapouriser to smoke cannabis may reduce pulmonary complications.^16^ Otherwise, there have been no peer-reviewed studies examining the effects of differing methods of smoking cannabis on lung health .

Cannabis and tobacco tend to be smoked differently. Typically, cannabis is smoked without a filter, to a shorter butt length, and the smoke is at a higher temperature. Furthermore, cannabis smokers inhale more deeply, hold their breath for longer and perform a Valsalva manoeuvre at maximal breath-hold.^17–19^

Some smokers make joints with marijuana leaves alone,^20^ whereas others usually smoke ‘spliffs’ containing both cannabis and tobacco leaves.^21^ This varies from one country to another. Cannabis users are also more likely to concurrently smoke tobacco, separately, making it difficult to disentangle the effects of cannabis itself on the lungs.

### Effects of tobacco smoking

The effects of cigarette (tobacco) smoking on lung health are well known. They include symptoms of cough, sputum production (which when marked constitutes chronic bronchitis), wheeze and shortness of breath. Spirometric changes such as a progressive, largely irreversible decrease in forced expiratory volume in 1 s (FEV_1_) and FEV_1_ divided by forced vital capacity (FVC)−FEV_1_/FVC ratio occur. This ratio is the most reliable measure of airflow obstruction. These changes may be accompanied by air-trapping within the lungs, measured physiologically (as increased total lung capacity (TLC) or residual volume (RV) or the ratio RV/TLC) or radiologically, on chest X-ray, etc. Chronic persistent, progressive airway narrowing, damage to the alveoli (emphysema) and effects on small airways (investigated with more sophisticated methods, beyond the scope of this review), in varying proportions, comprise the condition chronic obstructive pulmonary disease (COPD). As cannabis was thought to affect the lungs in similar ways to tobacco, it was logical to use spirometric measurements to detect the adverse health effects of marijuana. However, with concurrent tobacco smoking, it is difficult to separate changes due to cannabis from those due to tobacco.

The general paucity of data, the evolving nature of available marijuana (newer, stronger forms, modes of inhalation, etc) and the important confounding factor of tobacco use have led to different interpretations of the health impact of marijuana by the public as well as within the research and medical community. The purpose of this review is to assess current knowledge of the chronic effects of cannabis smoking on respiratory function and the progression of pulmonary disease as well as to identify potential directions for future research.

## Methods

MEDLINE/PubMed (NLM), Scopus (Elsevier) and Science Citation Index Expanded (Web of Science) databases were searched for English-language peer-reviewed articles from 1 January 1968 to 6 June 2015. These were refined by the following search terms: ‘marijuana smoking lung’, ‘cannabis smoking lung’, ‘marijuana smoking pulmonary’ and ‘cannabis smoking pulmonary’. This method yielded 256 articles.

These initial 256 results were reduced to 114 peer-reviewed articles, as the other 142 were not published in peer-reviewed journals. The remaining 114 articles were individually screened by title and abstract, looking for measurements of pulmonary function and long-term cannabis use in humans. Case reports and case series were omitted. The final count contained 19 articles fitting all the above criteria. Each article was then individually appraised, with the main findings and conclusions tabulated.

An additional search was also conducted for English-language peer-reviewed articles, as above, containing the following search terms: ‘cannabis bullous lung’, ‘cannabis bullae lung’, ‘cannabis pneumothorax’ ‘bong lung’, ‘marijuana bullous lung’, ‘marijuana bullae lung’ and ‘marijuana pneumothorax’. This yielded 69 articles, which were individually screened by title and abstract for articles relating to bullous lung disease in marijuana smokers. Case reports and case series were included in this search, and the final count contained 18 articles.

## Results

### Simple lung function measurements

The results of these 19 studies are summarised in Table 1. A total of 11 studies were cross-sectional,^20,22–31^ and 8 were observational cohort studies.^21,32–38^ Eighteen out of 19 studies included spirometric measurements in chronic marijuana smokers.^20–29,31–35,37–39^ The results from these studies varied; eight studies found no significant changes in FEV_1_/FVC ratio,^23,24,26–29,32,35^ whereas six studies found a significant decrease in FEV_1_/FVC in chronic marijuana-only smokers compared with that in controls, with 0.5–1.9% reduction.^20–22,34,37,38^ The remaining studies also varied in their findings, but suggested that simply measuring the FEV_1_/FVC ratio does not accurately reflect the pulmonary effects of chronic marijuana use.

All studies reporting a significant decrease, ~1.5%, in FEV_1_/FVC ratio in marijuana smokers published incomplete data; in particular, most omitted the absolute results for FVC alone. However, two of these studies reported no significant effect on FEV_1_ in chronic cannabis use.^20,22^ Sherrill *et al.*^34^ in a follow-up survey of a random, stratified, cluster sample of the Tucson population, aged 15–60 years (*n*=856 who had at least two measurements), found a reduction in FEV_1_/FVC of −1.9±0.7% and in FEV_1_ of −142±44 ml only in previous marijuana smokers, with a nonsignificant decrease of −0.5±0.6% in current marijuana smokers.^22^ Surprisingly, in 1,239 of the same subjects, tested on at least one occasion, FEV_1_/FVC was reduced by 0.5±0.6% and FEV_1_ increased by 58 ml. However, in a population-based cohort born in 1972 and 1973 in Dunedin, New Zealand (*n*=1,037), Hancox *et al.* showed no significant association between chronic marijuana use and change in FEV_1_ or FEV_1_/FVC at age 32 but found an increased FVC.^32^ Subsequently, both Pletcher *et al.*, in a longitudinal study over 20 years, recruited in 1985, examining Coronary Artery Risk Development In 5115 young Adults, and Kempker *et al.*, in a cross-sectional study of 7,716 US adults from the National Health And Nutrition Examination Study cohort 2007–2008 and 2009–2010 surveys, reported similar findings as had Tilles *et al.* previously, although with a much smaller sample size (*n*=15 women).^25,31,33^ Pletcher *et al.*, reported that FEV_1_ and FVC were 36 and 59 ml greater in cannabis users, with >10 joint-years’ smoking history, than in non-smoking controls.^33^ Kempker^27^ showed no effect on FEV_1_/FVC up to 20 joint-years but over 20 joint-years was associated with a 2.1-fold risk for FEV_1_/FVC ratio <70%, accounted for by a significant increase in FVC and no significant reduction in FEV_1_.^25^ Interestingly, Tashkin *et al.*^29^ reported a nonsignificant increase in both FEV_1_ and FVC in a convenience sample of 144 heavy marijuana smokers compared with other smoking groups, and in another study, in the same subjects,^39^ found that heavy habitual marijuana use, over a period of 8 years, was not associated with a decline in FEV_1_. Taken together, this information suggests that, although in some cases FEV_1_/FVC decreases by ~1.5% in chronic users, this may relate more to an increase in FVC rather than to a reduction in FEV_1_. This represents a major difference from the effects of tobacco smoking. The possibility that the effect on FEV_1_ is due to selection of people with higher FEV_1_ (because those with lower values do not smoke or do not continue to smoke cannabis) cannot be excluded in some of the studies.

### Effects of dose and duration of exposure

Four studies found a dose-related response to marijuana exposure.^20,21,25,33^ Using ANCOVA, Aldington *et al.* (*n*=75 with a mean of 54.2 joint-years) found that, for every joint-year of smoking, there was a decrease in FEV_1_/FVC of 0.019%. Changes due to chronic cannabis use (per joint-year) were also found in specific airway conductance (−0.0017%), functional residual capacity (+0.0013%) and TLC (+0.002%) but not in FEV_1_ (unfortunately FVC was not reported). From this, they estimated that one pack-year of tobacco was equivalent to 4.1–7.9 joint-years of cannabis smoking (alternatively 1 joint of cannabis was equivalent to 2.5–5 cigarettes) in causing airflow obstruction.^20^ This was echoed by MacLeod *et al.*, who found a 0.3% increase in the prevalence of COPD (defined by FEV_1_/FVC<0.70) for each additional joint-year in marijuana and tobacco concurrent smokers (*n*=252) in their sample of 500 subjects recruited from a general practice in Edinburgh, Scotland. It is important to note that subjects in this study were eligible for recruitment only if they reported significant tobacco or cannabis use, defined as at least 5 pack-years and/or 1 joint-year, with none of their participants smoking exclusively cannabis.^21^ Contrary to previous studies, Pletcher *et al.* (*n*=795 in Coronary Artery Risk Development In 5115 young Adults) noted that lifetime marijuana exposure was associated with an increase in FEV_1_ up to 7 joint-years with a decline thereafter at a slope of −2.2 ml/joint-year, although FEV_1_ was still nonsignificantly higher than in controls at all exposure levels (by 36 ml at >10 joint-years lifetime exposure).^33^ FVC, even after 20 joint-years, was still significantly raised by 59 ml. In both cases, however, FVC and FEV_1_ had a non-linear relationship with marijuana, which again differs from that of tobacco smoking.^33^ Kempker *et al.* calculated in 855 cannabis smokers that, for each additional joint-year smoked, there was no significant change in FEV_1_ %predicted (0.02+0.02), whereas FEV_1_/FVC decreased by −0.03±0.01% (*P*=0.02), accounted for by a significant increase in FVC 0.07+0.02% (*P*=0.004).^25^

There is evidence that current use of marijuana may influence spirometric measurements. Sherrill *et al.*^34^ reported that only former cannabis smokers (*n*=856) had decreased FEV_1_/FVC ratios, with no significant change in current smokers. In support, Kempker *et al.* found that use of cannabis in the past month was associated with an increased FVC for each additional day, with no FEV_1_ decrease.^25^ Eight of the 19 studies reported no criteria for abstinence from marijuana before spirometric testing.^21,22,24,34,37–39^ Strictly this confounds interpretation, making it difficult to distinguish acute from chronic effects of marijuana as acute bronchodilator effects of d-9-THC can be seen 2–3 h after inhalation^6,40^ and up to 6 h after oral ingestion.^5^

### Other measures of lung function

Very few studies have examined more sophisticated measurements of lung physiology. Aldington *et al.* found a very small increase in plethysmographic TLC of 0.14 l in cannabis users in a convenience sample from the Greater Wellington region, New Zealand, which was supported by similar findings by Hancox *et al.*, who reported an increase of just 0.03 l with an increase of 0.01 l in functional residual capacity and RV.^32^ Tilles *et al.* also found that marijuana smokers (*n*=15) had a TLC of 108±15% of predicted, which was significantly raised.^31^ Increased RV may indicate early signs of small airway dysfunction and air-trapping; however, it is not a very specific measure of small airway disease.^41^ Conversely, results from Tashkin *et al.*^28,29^ (*n*=74 and *n*=144) showed no significant changes in multiple measures of small airway function (FEV_25–75%_, closing volume, closing capacity, RV) between chronic cannabis smokers and non-smoking controls.

Three studies also reported an association of chronic cannabis smoking with increased airway resistance (0.03–0.38 cm H_2_O/l/s)^28,29,32^ and four studies found reduced specific airway conductance (0.007–0.07 ml/s/cm H_2_O/l).^20,28,29,32^ There was no association of these changes with change in lung volume.^32^ Central airway secretions or inflammation or oedema would be a potential explanation.

In studies measuring carbon monoxide transfer factor (TLco), two small studies (*n*=28 and *n*=15) found a significant decrease in TLco with chronic concurrent marijuana and tobacco use down to 65±15% of predicted.^27,31^ However, these studies did not find a significant reduction in TLco with marijuana-only use. Three other larger studies also found no significant decrease.^20,29,32^

Airway responsiveness was measured in three studies.^24,30,38^ Hernandez *et al.* studied 23 subjects from Texas, including six marijuana smokers, using histamine to measure bronchial responsiveness but found no difference in PD_50_ sRaw (dose of histamine causing a 50% increase in specific resistance, sRaw) in marijuana smokers compared with controls, whereas asthmatics showed significant hyper-reactivity to histamine. Larger studies by Tashkin *et al.*, in a convenience sample of 113 out of 542 subjects, and by Taylor *et al.*, in a sample of cannabis-dependent 21-year olds (*n*=91), from 943 members of the Dunedin birth cohort (*n*=1037), measured metacholine responsiveness as the provocative dose causing a 20% reduction in FEV_1_ (PC_20_). They found no significant difference in the number of marijuana-only smokers who had a positive PC_20_ metacholine compared with that in non-smokers. However, it is important to note that, after logistic regression to control for the effect of tobacco and cocaine, Tashkin *et al.* found that concurrent smokers of marijuana and tobacco had a significant association with hyper-responsiveness to metacholine.

The relationship between tobacco and marijuana together on lung function is complex and somewhat controversial. Sherman *et al.* reported a reduction in FEV_1_/FVC and TLco in a marijuana and tobacco-smoking group (*n*=13) but no change in the marijuana-alone group (*n*=19).^27^ Tan *et al.* in a sample of 878 people over 40 years of age in Vancouver, Canada, also found a significant increase in respiratory symptoms in the marijuana and tobacco-smoking group but not in the marijuana-only group.^35^ However, three other studies have reported no additive effects on lung function of chronically smoking both marijuana and tobacco.^30,37,39^

### Respiratory symptoms

Eight out of nine studies, which quantified respiratory symptoms, reported an increase in respiratory complaints with odds ratios (ORs) of up to 2.98 compared with non-smoking controls.^20–22,26,28,29,34,38^ Respiratory symptoms recorded included increased incidence of cough, sputum production, shortness of breath and wheeze. Taylor *et al.* also reported significant increases in exercise-related shortness of breath (OR 1.65), nocturnal wakening with chest tightness (OR 1.72) and morning sputum production (OR 2.44) in a birth cohort of 1,037 subjects at 21 years of age.^38^ These ORs were even higher if casual cannabis users were excluded. However, in a cross-sectional study of 6,728 subjects gathered from the National Health And Nutrition Examination Study survey, which was conducted between 1988 and 1994, there was no association with shortness of breath (OR 1.29, *P*=0.26) compared with non-smoking controls after adjusting for age, although they did find an increase in cough, phlegm, wheezing and chronic bronchitis symptoms with similar OR to tobacco users, which is interesting as in this sample marijuana users were, on average, 10 years younger than tobacco smokers.^26^ Marijuana users (*n*=414) were included in the sample if they had smoked more than 100 cannabis cigarettes in total and had at least 1 day of use in the previous month; they were not asked about the frequency of use or overall exposure above 100 cigarettes. In addition to these nine studies, Hancox *et al.* also found an association between cannabis and morning cough, sputum production and wheeze compared with non-smokers.^42^

It was observed that cannabis smoke produces large airway epithelial damage, oedema, erythema and increased secretions with goblet cell hyperplasia,^43^ loss of ciliated epithelium and squamous metaplasia on biopsy. There is also evidence of additive bronchial epithelial damage in combined cannabis and tobacco smokers.^44^

### Effect of quitting cannabis smoking

Examining the effect of quitting is another approach to determining the effect of smoking cannabis. The effect of quitting cigarette (tobacco) smoking is well known.^45–47^ Unfortunately, there are only two studies available of quitting cannabis. Tashkin reported that in a convenience sample of 299 young adults, including 95 regular cannabis-only smokers, 71 cannabis and tobacco concurrent smokers and 49 tobacco-only smokers, those who became non-smokers of both substances had no increased risk for chronic bronchitis compared with never smokers at a mean of 9.8 years of follow-up.^29,48^ However, those who continued smoking either substance had an increased risk for chronic bronchitis over the follow-up period. Hancox *et al.* analysed data from a well-characterised, population birth cohort of 1,037 subjects followed from birth to age 38 years with symptomatic assessment at 18, 26, 32 and 38 years of age.^42^ Frequent cannabis users numbered 157, 162, 138 and 99 compared with 540, 487, 608 and 703 non-users at different time points. There were significant reductions in morning cough, sputum production and wheeze, but not shortness of breath on exertion in the 26, 52, 54 and 50 quitters compared with continuing smokers, whether of cannabis alone or cannabis and tobacco. Furthermore, symptoms in the quitters were reduced to levels similar to those in never users. Sherill *et al.* reported that the risk for respiratory symptoms in previous non-tobacco smokers reduced to normal after quitting, but a significant reduction in FEV_1_, FEV_1_/FVC and Vmax_50_ persisted in previous cannabis smokers, although it was not present in current cannabis smokers.^34^

### Bullous lung disease and emphysema

Characteristic, peripheral, cystic changes on high-resolution computed tomography scan are often found in patients who are (probably heavy) cannabis smokers,^49^ although the specificity and rate of occurrence of these findings are unclear. Only one cross-sectional study, recruited from the Greater Wellington area, measured radiological changes, reporting an increase in rates of macroscopic emphysema in tobacco smokers (16% in tobacco±cannabis *n*=183), but not in cannabis-only smokers (1.3% of 75).^20^ However, the cannabis smokers showed a significant increase in low-density lung regions compared with tobacco smokers, and this was interpreted as a result of airflow obstruction and hyperinflation rather than microscopic emphysema.

Aside from the above-mentioned study, 7 case series and 10 case reports were also found, including a total of 56 marijuana smokers presenting with bullous lung disease (often with pneumothorax) with concurrent tobacco smoking present in all but 3 cases. The results of these studies are summarised in Table 2. One case report was excluded because of being included in a subsequent case series.^50,51^ The majority of subjects in these cases were heavy smokers of marijuana, although it is quite difficult to compare usage as the units of measurement varied. Seven case series and six case reports had predominantly upper lobe involvement (2 with Vanishing Lung Syndrome, VLS), and one case report had predominantly lower lobe bullae. For those with upper lobe involvement, the majority had peripheral emphysema. Lung function was measured in four case series, with the majority of cases having normal lung function results.

### Cannabis and lung cancer

Currently, the evidence regarding an association of cannabis smoking and lung cancer is inconclusive.^52,53^ Some studies have found precancerous histological changes in bronchial biopsies of marijuana smokers.^44,54^ However, epidemiological studies have not found a definite association. A pooled analysis of 6 case–control studies with a total of 2,159 lung cancer cases and 2,958 controls found little or no association between cannabis smoking and lung cancer; the overall pooled OR was 0.96 for habitual versus non-habitual smokers.^55^ Another large retrospective cohort study of 64,855 subjects found no increased risk for cancer after 8.6 years, although their subjects were relatively young even after follow-up.^56^ Other epidemiological studies have reported associations with lung cancer with heavier marijuana use. A 40-year longitudinal cohort study in 49,321 Swedish conscripts found that those who smoked cannabis >50 times had a twofold risk of developing lung cancer.^57^ Although this study is large, there are significant flaws in its methodology that limit the conclusions that can be drawn. The subjects were only assessed for tobacco and cannabis use at the time of conscription with no information on use patterns before conscription and the 40 years after conscription. A small case–control study in 403 subjects (79 lung cancer patients including 14 heavy cannabis users) also found, after adjusting for tobacco, an increased risk for lung cancer for each joint-year smoked, although not in the 2 lowest tertiles.^58^ However, owing to the small number of cases (*n*=14) and controls (*n*=4), it is highly likely that relative risk estimates are inflated.

## Discussion

Although there are clear inconsistencies between these 19 studies, which may relate to subjects studied as well as methodology, we can nevertheless draw various conclusions regarding the effects of cannabis on lung function. Cannabis smokers usually also smoke tobacco, either concomitantly or separately. This makes it difficult to disentangle the effects of the two. Quantification of cannabis use compounds the difficulty of dose–response studies. Although studies consistently show the effects of cannabis on symptoms (chronic bronchitis), there are mixed reports regarding airflow obstruction. The larger cross-sectional and observational cohort studies have found no association with reduced FEV_1_/FVC. Self-selection (where subjects with a tendency to airway narrowing reject cannabis smoking) cannot be excluded. However, more recent, larger studies have found that chronic cannabis users have an increased FVC alone, or in conjunction with an increased FEV_1._^32,33^ A larger rise in FVC compared with FEV_1_ could also contribute to previous observations of a decreased FEV_1_/FVC ratio.^33^

This contrasts with the reduced FEV_1_ and FEV_1_/FVC ratio (indicating airflow obstruction) consistently associated with cigarette smoking and COPD. The cause of the raised FVC in chronic marijuana smokers is unclear. It has been suggested that it might be because of training of respiratory muscles by the characteristic inhalation techniques employed by marijuana smokers.^19,59,60^ However, other evidence that training can increase FVC by this amount in humans is scant.^61^ Bronchodilatation of small airways can increase FVC, e.g., by up to 300 ml or 8.6% of predicted after inhaled salbutamol 100 μg.^62^ An acute bronchodilator effect of cannabis is well described; in three studies, marijuana was shown to increase FEV_1_ by 150–250 ml above baseline.^6^ Relative preservation of FEV_1_ (36 ml greater in marijuana smokers, with >10 joint-years of exposure, compared with that in controls) as found by Pletcher could relate to a bronchodilator effect, as suggested by Kempker *et al*.^25^

The lack of a defined marijuana abstinence period complicates interpretation of spirometric results. Current cannabis smokers had higher FVC values in studies of Kempker^25^ and Sherrill *et al.*^34^ Against this, tachyphylaxis to the bronchodilator effect would be anticipated, and in studies in asthmatics acute bronchodilatation lasted only about 2–6 h. The small but consistent increases in airway resistance, and reduction in airway conductance,^28,29,32^ suggest small effects on central airways, which may be explained by airway secretions, inflammation or oedema.

Another speculative, potential explanation for the absence of chronic airflow obstruction with cannabis smoking may relate to the well-documented anti-inflammatory and immunomodulatory effect of THC^63^—e.g., impairment of functional activity of stimulated alveolar macrophages (antimicrobial and respiratory burst, impaired cytokine production and nitric oxide production)^27,64–66^ thought to be critical in COPD pathogenesis.^67–69^

The clinical relevance of all this is unclear. COPD resulting from an inflammatory response in the airways to tobacco smoking is a major epidemic, currently the sixth leading cause of death worldwide and projected to be the fourth leading cause of death by 2030. Chronic marijuana smokers, who often also smoke tobacco, present with similar chronic respiratory symptoms but do not appear to develop airflow obstruction and COPD.

### Future studies

In general, more information is required on cannabis smoking and the lung. The difficulties with joint-years as the measure of exposure have been mentioned. The observation that chronic marijuana smokers generally buy and keep track of their supply in grams suggests that grams per year may have advantages for both clinicians and researchers. It has been reported that the amount of cannabis purchased each month, and the intensity of the high afterwards, predicted respiratory symptoms independently of frequency of use.^70^ Although this will not overcome the problems with self-reporting illegal substance use, it may serve as a better measure of standardisation than joint-years.

Further research is necessary to clarify the relationship between respiratory symptoms and lung function. Further studies are needed to address acute bronchodilatation as a possible confounding factor in studies on chronic airflow obstruction. Serial examination of the bronchodilator effect of cannabis to look for tachyphylaxis would be of interest. More research into the effects of cannabis on the pathogenesis of COPD in relation to small airway inflammation, cytokine production and macrophage involvement is needed as well.

Other measures of airflow obstruction, including airway resistance, plethysmographic lung volumes and particularly measurements of small airways function, including imaging studies, are required. The priority is to understand why tobacco and cannabis smoking both cause chronic bronchitis yet have different effects on lung physiology. The pharmacological and pathophysiological basis of this needs to be established.

More work is needed in quitters of cannabis smoking, including motivations to quit, the effect on respiratory symptoms and lung function and bronchoscopic biopsy studies, to examine the effects on goblet cell hyperplasia and other histological findings.

## Conclusions

This review clearly shows that chronic marijuana smoking is associated with respiratory symptoms and increase in FVC. The mechanisms for these effects and the differences from the effects of tobacco remain unclear. More work needs to accurately measure cannabis use as well as measure all aspects of respiratory health, particularly breathlessness and exercise tolerance. More importantly, however, there needs to be larger, longer-term studies with marijuana smokers who do not smoke tobacco.

There is clear evidence that marijuana causes similar symptoms to tobacco smoking (chronic bronchitis) and produces similar large airway pathological features. There is some evidence that the combination of tobacco and marijuana is additive. Tobacco unequivocally causes chronic airflow obstruction and COPD but only in a minority of smokers. Cannabis smoking, however, produces an increase in FVC and the reason(s) for this are unclear and require elucidation. Taking a more detailed history with regard to cannabis smoking and other illicit inhalational drugs should be part of the standard respiratory assessment of all patients, which would also support better epidemiological data collection for future studies, particularly in the primary care population.

## Figures and Tables

**Table 1:** Summary of studies on the association between chronic cannabis use and lung function

*Author (year)*	*Subjects (*n*)*	*Tobacco control*	*Results*
Cruickshank^23^	60	No	No significant differences found between cannabis smokers and control with respect to FEV_1_ and FVC.
Tashkin *et al.*^28^	74	Yes	No differences in spirometry results compared with those in matched and unmatched controls. A significant increase was noted in Raw and decrease in sGaw compared with those in both controls. Although not signficant, FVC was raised compared with that in both controls.
Hernandez *et al.*^24^	23	No	Spirometry results of marijuana smokers were not significantly different from those of controls. There was also no significant difference in bronchial reactivity to histamine compared with controls.
Tilles *et al.*^31^	68	Yes	Cannabis smoking with or without tobacco smoking was associated with a reduction in TLco (74% predicted±20%, *P*<0.05). In marijuana and marijuana plus tobacco smokers, both FEV_1_ and FVC were significantly increased compared with that in non-smokers.
Bloom *et al.*^22,^^a^	990	Yes	There was a significant increase in respiratory symptoms of phlegm and wheeze, but not cough or shortness of breath, in non-tobacco cigarette smokers whether or not they had ever smoked tobacco cigarettes. There was a significant decrease in FEV_1_/FVC compared with that in controls. There was no significant change in FEV_1_ in any non-tobacco-smoking category.
Tashkin *et al.*^29^	446	Yes	Smokers of marijuana and/or tobacco had significantly increased rates of chronic cough, wheeze and sputum production. There was an increase in Raw and decrease in sGaw in male marijuana smokers but not in tobacco smokers. FEV1 and FVC of marijuana smokers were not significantly different from those of controls.
Sherrill *et al.*^34^	856	Yes	Non-tobacco smoking was associated with chronic cough (OR=1.73), chronic phlegm (OR=1.53) and wheeze (OR=2.01). There was a significant reduction in FEV_1_ and FEV_1_/FVC ratios with previous non-tobacco smoking but not with current smoking.
Sherman *et al.*^27^	63	Yes	Macrophage oxidant release, small airway integrity and alveolar gas exchange were similar in both non-smokers and marijuana smokers. There was no significant difference in lung function measurements between marijuana-only smokers and non-smokers. Marijuana and tobacco concurrent smokers showed a decrease in FEV_1_/FVC and TLco.
Tashkin *et al.*^30^	542	Yes	No significant difference in AHR to metacholine was found in non-smokers and marijuana smokers without tobacco. Logistic regression showed a significant response to metacholine with marijuana smoking, however. No dose–response relationship was found between AHR and lifetime marijuana use.
Tashkin *et al.*^36^	394	Yes	Although tobacco smoking showed an FEV_1_ decline in men, marijuana smoking was not associated with FEV1 decline in either gender.
Taylor *et al.*^38,^^a^	1037	Yes	After controlling for tobacco, cannabis users had an increase in wheezing, exercise-related shortness of breath, nocturnal wakening with chest tightness and morning sputum production (*P*<0.05). Cannabis users had decreased FEV_1_/FVC compared with non-smokers. There was no significant increase in AHR in tobacco or cannabis users.
Taylor *et al.*^37,^^a^	1037	Yes	After stratifying by use of cannabis, at each age increasing cannabis use was associated with a decline in FEV_1_/FVC. After adjustments for other co-variates, cannabis as a predictor was only marginally significant (*P*<0.09).
Moore *et al.*^26^	6728	Yes	Marijuana use was significantly associated with chronic bronchitis symptoms, coughing on most days, phlegm production, wheezing and chest sounds without a cold. After adjustment for confounders, cannabis smoking was not associated with an FEV_1_/FVC ratio <70% (*P*=0.99).
Aldington *et al.*^20^	339	Yes	Both cannabis and tobacco-smoking groups showed a reduction in FEV_1_/FVC. Tobacco reduced FEV_1_, whereas cannabis smoking had no effect on FEV_1_. Tobacco smoking was associated with macroscopic emphysema by CT, but not cannabis-only smoking.
Tan *et al.*^35^	878	Yes	Marijuana-only smokers had no significant increase in risk for COPD as defined by symptoms and spirometry. However, concurrent use of tobacco and marijuana produced an increased risk for respiratory symptoms and COPD.
Hancox *et al.*^32^	1037	Yes	After adjustment for tobacco, cannabis exposure was associated with increased FVC and TLC, but there was no significant association with FEV_1_ or FEV_1_/FVC. Cannabis was associated with increased Raw and lower sGaw.
Pletcher *et al.*^33^	5119	Yes	Marijuana exposure was non-linearly associated with lung function, unlike tobacco (*P*<0.001). Lifetime marijuana exposure showed an increase in FEV_1_ over time at up to 7 joint-years and declining thereafter. FVC was significantly elevated even in heavy users up to 20 joint-years (*P*<0.001). Both FEV_1_ and FVC were increased at all exposure levels compared with those in controls.
Kempker *et al.*^25^	7716	Yes	For cannabis smokers with 1–5 and 6–20 joint-years, there was no association with an FEV_1_/FVC<70% (OR=1.1). Those with over 20 joint-years did (OR=2.1). Use of marijuana in the past month was associated with increased FVC (0.13±0.03%, *P*=0.0001) for each additional day but no decrease in FEV_1_.
Macleod *et al.*^21^	500	Yes	Cannabis and tobacco use together was associated with increased cough, sputum production and wheeze. After adjustment for tobacco use, age, gender and deprivation, each additional joint-year of cannabis was associated with 0.3% increase in the prevalence of FEV_1_/FVC<70%.

Abbreviations: AHR, airway hyper-responsiveness; CT, computerised tomography; COPD, chronic obstructive pulmonary disease; FEV_1_, forced expiratory volume in 1 s; FVC, forced vital capacity; sGaw, specific airway conductance; Raw, airway resistance; TLco, transfer factor of the lung for carbon monoxide.

a Hancox *et al*.: a follow-up of two studies by Taylor *et al.*^37,^^38^; Sherrill *et al*.^34^: a follow-up of a study by Bloom *et al*.^22^

**Table 2:** Summary of case studies and case series on the association between cannabis smoking and bullous lung disease

*Author (year)*	*Subjects (*n*)*	*Mean age*	*Marijuana smoking*	*Tobacco smoking (pack-years)*	*Results*
Feldman *et al.*^71^	1	24	14–28 g/week for 10 years	14	Spontaneous pneumothorax. Microscopy showed ruptured bulla, serosal adhesions and focal atelectasis
Johnson *et al.*^49^	4	38	2 joints/week to 3 joints/day	3–15	Bilateral upper zone peripheral bullae in all four cases. One with paraseptal and two with apical bullous emphysema
Rawlins *et al.*^72^	2	29	Yes^a^	Yes^a^	Bilateral giant lung bullae and severe upper lobe emphysema
Thompson *et al.*^73^	3	39	'Moderate' for 10 years to 'heavy' for 24 years	9–20	Large upper lobe bullae
Phan *et al.*^74^	1	26	10 pipes a day for 5 years	1	Bilateral cystic and bullous changes in lower lobes. Microscopy showed fibrosis and macrophage infiltration
Beshay *et al.*^75^	17	27	53 joint-years	0–25	Multiple apical bullae or bullous emphysema in upper lobes. Histology showed macrophages
Hii *et al.*^51^	10	41	11–149 joint-years	1–27	Asymmetrical bullae peripherally and centrally in upper and mid zones.
Reece^76^	1	56	10 cigarettes per day for 25 years	>1	Mixed tobacco and cannabis in joint. Multiple giant lung cysts on CT scan, no lobe predominance.
Gao *et al.*^77^	1	23	Yes^a^	None	Cystic fibrosis. Bilateral large upper lobe bullae. Recurrent pneumothorax
Allen^78^	1	18	1 oz weekly for 4 years	3	Bilateral apical bullae up to 3 cm. Histology showed emphysematous changes with pigmented macrophages and DIP-like changes.
Shah *et al.*^79^	1	27	‘Heavy’ use for 10 years	20	Large left apical bulla and right apical blebs. CT scan following chest drain of pneumothorax
Sood *et al.*^80^	1	33	‘Off and on’ for 10 years	15	VLS on the left side shown on chest X-ray and CT scan
Gargani *et al.*^81^	2	41	Yes^a^	NA to 39	One patient had left apical bullae, the other had right upper and middle lobe bullae. In both patients, one bulla contained *Aspergillus*
Golwala^82^	1	25	24 joint-years	1	Bilateral bullae with upper lobe predominance. Previous untreated sarcoidosis but no current clinical/radiological features
Tashtoush *et al.*^83^	1	65	‘Heavy ’ use for 20 years	None	Poorly controlled AIDS and previous IV heroin use. Bilateral large lung bullae characteristic of VLS
Fiorelli *et al.*^84^	8	30	7 joints per week to 6 joints per day	15–40	Eight of 13 marijuana smokers with spontaneous pneumothorax had bullae on CT scan. Six had paraseptal bullae and two had upper lobe involvement
Cary *et al.*^85^	1	48	86 joint-years	25	Bilateral upper and mid zone bullous disease. Air fluid level seen on left lung bulla. Sputum grew only Candida; no clinical signs of infection.

Abbreviations: CT, computerised tomography; DIP, desquamative interstitial pneumonia; IV, intravenous; VLS, vanishing lung syndrome.

a Undocumented amount.
